# Application and Design of Switches Used in CAR

**DOI:** 10.3390/cells11121910

**Published:** 2022-06-13

**Authors:** Paweł Głowacki, Piotr Rieske

**Affiliations:** 1 Department of Tumor Biology, Chair of Medical Biology, Medical University of Lodz, Zeligowskiego 7/9 St., 90-752 Lodz, Poland; 2 Department of Research and Development Personather Ltd., Inwestycyjna 7, 95-050 Konstantynow Lodzki, Poland

**Keywords:** CAR (Chimeric Antigen Receptor), safety switches, remote controlling, molecular switches, switchable CAR, CAR-T control, CRS, on-target off-tumor attack, CAR-T side effects

## Abstract

Among the many oncology therapies, few have generated as much excitement as CAR-T. The success of CAR therapy would not have been possible without the many discoveries that preceded it, most notably, the Nobel Prize-winning breakthroughs in cellular immunity. However, despite the fact that CAR-T already offers not only hope for development, but measurable results in the treatment of hematological malignancies, CAR-T still cannot be safely applied to solid tumors. The reason for this is, among other things, the lack of tumor-specific antigens which, in therapy, threatens to cause a lethal attack of lymphocytes on healthy cells. In the case of hematological malignancies, dangerous complications such as cytokine release syndrome may occur. Scientists have responded to these clinical challenges with molecular switches. They make it possible to remotely control CAR lymphocytes after they have already been administered to the patient. Moreover, they offer many additional capabilities. For example, they can be used to switch CAR antigenic specificity, create logic gates, or produce local activation under heat or light. They can also be coupled with costimulatory domains, used for the regulation of interleukin secretion, or to prevent CAR exhaustion. More complex modifications will probably require a combination of reprogramming (iPSc) technology with genome editing (CRISPR) and allogenic (off the shelf) CAR-T production.

## 1. Introduction

### 1.1. Nobel Prizes in Cell-Mediated Immunity Research and CAR

Immunotherapy of tumors is one of the most rapidly developing fields of medicine. Although it is associated with modern treatment, its origins can be found at the end of the 19th century. At that time, a case was described in which osteosarcoma regressed after contracting erysipelas. Shortly thereafter, William Bradley Coley made an effective attempt to treat tumors with the use of bacteria causing erysipelas [1]. Although this was largely chance-based, it exploited complex immunological mechanisms. Today, we have a much deeper understanding of immunology, which allows us to develop much more complex and effective cancer therapies. An example of the most advanced immuno-oncology therapy is the concept of chimeric antigen receptor (CAR). The classical CAR consists of an epitope-recognizing region, namely, a single-chain variable fragment (scFv), along with a costimulatory domain, most commonly CD28 or 4-1BB, and a CD3ζ signaling domain [2]. These genetically modified lymphocytes are used to destroy treatment-resistant blood tumors. However, for CARs to be designed, ample research over many years, was necessary. Among such research, the Nobel Prize-winning discoveries in the field of cellular immunity might be consider as some of the most important. Ralph M. Steinman (Nobel Prize 2011) discovered dendritic cells that, through the B7 protein, activate the CD28 domain present on T lymphocytes [3]. In CAR-T, CD28 became the classic chimeric receptor element [2]. Ralph M. Steinman shared the award with Bruce A. Beutler and Jules A. Hoffmann, whose work on Toll-like receptors, characteristic of macrophages, contributed to the use of the costimulatory molecule MyD88 (part of the TLR signaling pathway) as a strategy to increase CAR potency [4,5]. The discovery of macrophages and phagocytosis is attributed to Ilya Ilyich Mechnikov who, together with Paul Ehrlich received a Nobel Prize in 1908, made possible the use of CAR macrophages (CAR-M) as an alternative to CAR-T [6,7]. Another key factor was the observation of MHC-restricted antigen recognition, a phenomenon that makes T cell receptors (TCRs) require not only an antigen but also an MHC molecule to activate them (Doherty and Zinkernagel, 1996) [8]. The chimeric antigen receptor is designed to recognize antigens independently of the MHC, which greatly increases its effectiveness in killing, especially when the tumor cell lacks an MHC [9]. The MHC is also important in distinguishing between self and foreign cells. Sir Frank Macfarlane Burnet, along with Peter Brian Medawar, received awards in 1960 for understanding how cellular immunity, although effective against foreign antigens, does not destroy host tissues [10,11]. In addition, the practical application of knowledge of immunology in the first transplants performed by Joseph E. Murray and E. Donnall Thomas was important (awarded Nobel Prizes in 1990) [12,13]. In relation to CAR-T, these discoveries are particularly relevant because this therapy is an example of cell transplantation and can only be administered autologously. However, work is also underway on cells for allogeneic administration [14]. Another important line of Nobel Prize-winning research was on immune checkpoint blockade (James P. Allison and Tasuku Honjo in 2018). Receptors for PD-L1 are found, among others, on T cells, and serve to deactivate these cells. Tumors take advantage of this fact by increasing the expression of ligands for PD-1, thus disabling lymphocytes. The use of checkpoint inhibitors such as PD-L1 and CTLA-4 has shown suitable results in the treatment of many cancers. [15,16]. CAR-T can be subjected to modifications involving the knock-out of genes for checkpoint receptors, thus making them insensitive to tumor suppression without the separate addition of anti-PD-1 antibodies [17].

With these advantages, CARs have achieved undisputed success and have already been registered as a treatment for five blood malignancies. However, CAR therapy is being studied for more than just hematological malignancies and more than just T cells. Natural killer (NK) cells also seem to be attractive since they are not sensitive to suppression signals from Tregs. Additionally, natural killers are able to secrete tumor necrosis factor and interferon-gamma. NK cells are also attractive in the context of off-the-shelf therapies because they do not naturally cause graft-versus-host disease [18]. CARs have also been used for solid tumors such as glioblastoma, breast cancer, and prostate cancer [19,20,21]. Moreover, some studies indicate the possibility of using CAR-T to treat autoimmune diseases such as rheumatoid arthritis or lupus [22,23] and even to combat viral infections, including HIV infection and SARS-CoV-2 [24,25].

### 1.2. Dark Side of CAR Therapy

Despite the prevailing optimism, CARs also have a dark side, represented by potentially serious side effects. Among the more serious adverse effects that can lead to death are cytokine release syndrome (CRS), which may affect up to 92% of patients, and neurotoxicity (ICANS) [26,27,28]. In the case of solid tumors, the problem is also the lack of completely specific antigens, which makes possible a fatal on-target off-tumor attack with CAR-T application. In blood tumors, despite the relative specificity of the CD19 receptor, bone marrow aplasia can occur resulting in death or requiring stem cells transplantation [29,30]. Despite the developed treatment in the form of tocilizumab (an anti-IL-6 receptor antibody), there are cases where the patient dies regardless of its application [31,32]. Besides the mentioned tocilizumab, there is also treatment with siltuximab (anti-IL-6 antibody) [33]. As well as blocking the action of IL-6, IL-1 can also be blocked. Treatment with anakinra, which binds to IL-1, has shown good results in both ICANS and CRS contexts [34]. It is also possible to block IL-1 secretion at the macrophage level by blocking inflammasome formation [35]. Despite some achievements of this type of therapy, it is a symptomatic rather than causal treatment. To increase the possibility of limiting the side effects of therapy, especially on-target off-tumor attacks, a mechanism is needed to control the action of CARs. One potential method to enable elimination of the CAR-T in vivo could be the use of anti-CARs, namely other CAR-Ts, designed to kill previously administered lymphocytes [36]. However, this is an expensive solution that requires prior preparation of appropriate anti-CARs, as the long production of CARs prevents their rapid delivery within a reasonable time for the patient.

A solution to the problem of a lack of control may be the switches, which together with the CAR-containing construct, are implemented in lymphocytes. Thanks to their design, they make it possible to control the activity of CAR by administering appropriate molecules or even light or temperature. They enable the control of CAR-T activity in vivo, i.e., in the patient’s body, which means that lymphocyte deactivation can be ordered at the onset of symptoms or, in the case of the development of effective markers (e.g., MCP-1), even before the occurrence of complications [37]. Moreover, many of the developed switches, in addition to the mentioned function, can offer additional benefits due to their mechanism of action, as described further in this article.

**Figure 1:**
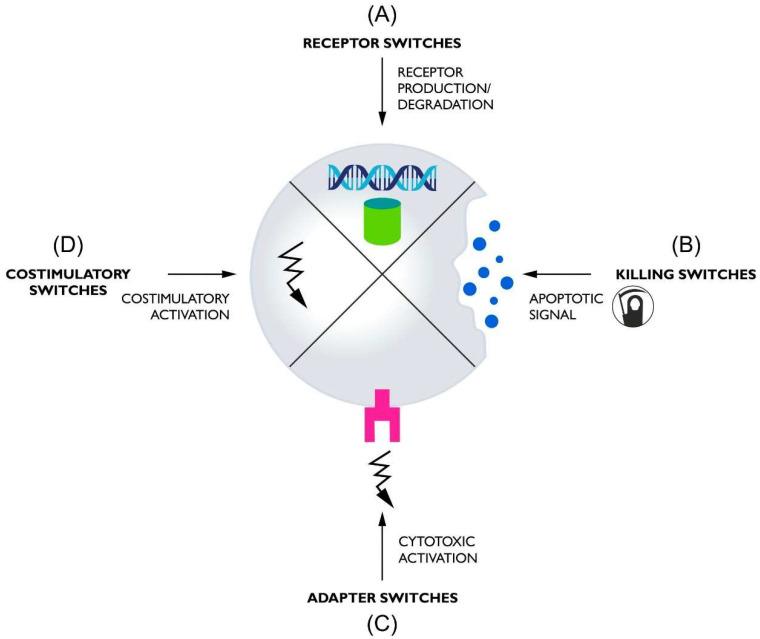
The diagram shows the general mechanism of the four classes of switches used to control CARs. (**A**) Receptor switches oversee the presence of the CAR protein in the cell by regulating its formation or elimination. (**B**) Killing switches control CAR-T activity by killing cells through apoptosis, triggered by a control molecule. (**C**) Adapter switches control receptor activation through a molecule mediating contact between CAR and the tumor antigen. (**D**) Costimulatory switches provide additional costimulation if appropriately activated.

Switches used in CAR-T can, based on their functions, be divided into four basic groups: (1) receptor switches that control the formation or degradation of CAR protein, (2) killing switches that regulate CAR-T activity by inducing lymphocyte apoptosis, (3) adapter switches that require a molecule linking CAR to antigens to function, and (4) costimulatory switches that provide an additional regulated amplifying signal (Figure 1).

## 2. Receptor Switches

Receptor switches are the largest and most complex group of switches. They can be divided into two minor subgroups: switches that regulate transcription and switches that modulate the rate of receptor degradation. As with all switches, receptor switches allow the control of CAR activity, but in this group, an additional benefit seems to be the reduction in the deleterious phenomenon of CAR-T exhaustion [38]. It is particularly important considering that strong CAR activation leads to cellular exhaustion and reduced therapeutic potential [39,40].

### 2.1. Degron Switches

Degron switches can be well described by the statement that their target is not CAR-T cells but only the CAR protein. They take advantage of the natural ability of cells to proteolyze proteins, in this case, CAR proteins.

**Figure 2:**
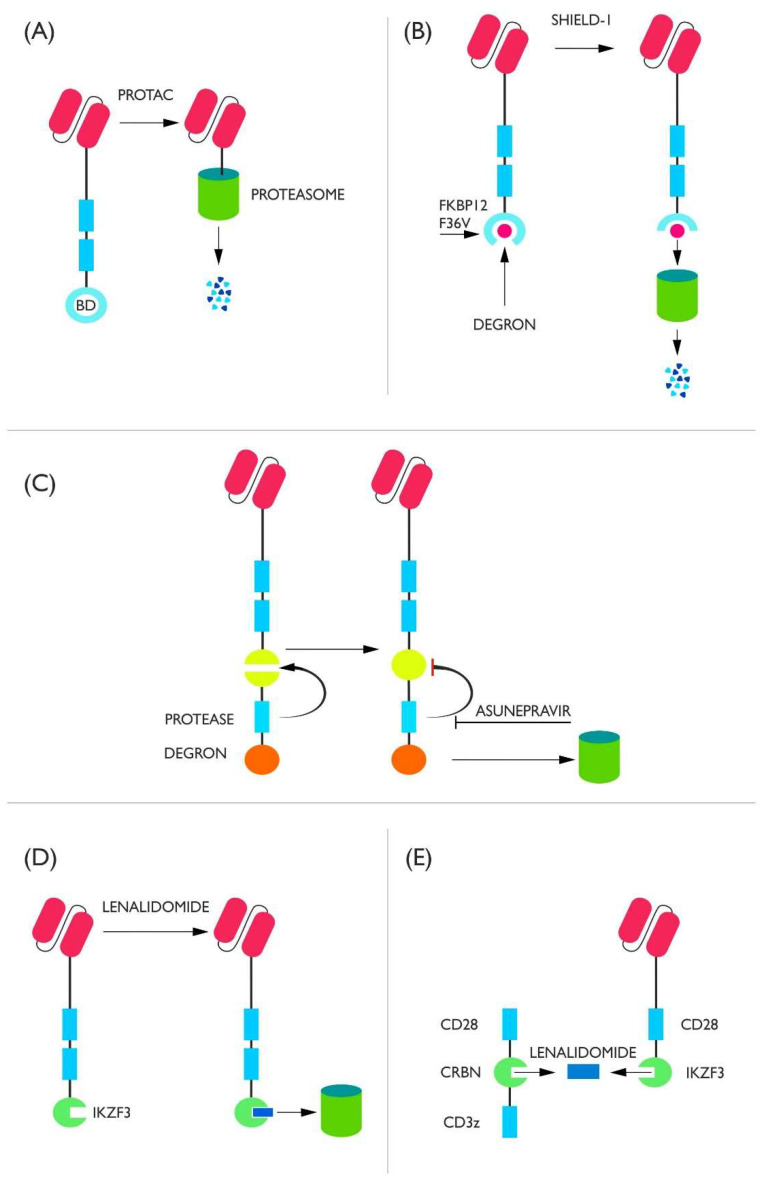
(**A**) The CAR protein is bound to the bromodomain. Upon delivery of the PROTAC compound, CAR attaches to the proteasome and is degraded. (**B**) The CAR protein is connected to a degron spatially covered by the FKBP12 F36V protein. Under the influence of the Shield-1, FKBP12 changes its conformation, exposing the degron, which leads to proteolysis. (**C**) CAR is linked to protease and a degron. Protease spontaneously cuts off the degron from the CAR protein so that it is not degraded. When asunepravir is administered, the protease is blocked and the whole CAR is destroyed in the proteasome. (**D**) Off switch. Addition of lenalidomine leads to proteasomal degradation. (**E**) On switch. Administration of lenalidomide causes two separate parts of the receptor to fuse into a whole and activates the receptor.

One representative of this class is the proteolysis-targeting chimera (PROTAC) compound-based system. The bromodomain (BD) is attached to the intracytoplasmic end of the CAR. Upon the addition of PROTAC compounds (ARV-771 or ARV-825), E3 ligase binding occurs, which ultimately leads to proteasomal degradation of the receptor. It is noteworthy that epoxomycin is able to block the degradation resulting from the use of PROTAC. As shown in this study, the use of PROTAC degrades the CAR protein and prevents the cell from continuing activity. This effect is reversible when PROTAC administration is discontinued, but some cells die (approximately 20–30%) as a result of the degradation of endogenous brd4 (Figure 2A) [41].

Another example of control by degradation is CAR-LID. The CAR protein is associated with FKBP12 F36V and a degron that is located in a way in which access to it is limited by its spatial conformation (cryptic degron). In order to expose the degron and allow proteolysis it is necessary to administer the Shield-1 compound, which makes the degron domains accessible to proteasomal proteins (Figure 2B). In vivo, the maximum down-regulation was 80% of CAR surface protein expression relative to lymphocytes not incubated with Shield-1. Unfortunately, this system cannot be universally applied; when FMC63 anti-CD19 scFv was used, surface CAR degradation occurred without the involvement of Shield-1, which means that some protein compositions can be degraded independently of the controlling molecule [42]. Shield-1 was also used to create a counteracting switch, which requires continuous administration of Shield-1 to prevent CAR degradation, while the chimeric receptor protein is degraded upon cessation of its introduction. The result is a combination of both counter-acting systems, such that Shield-1 administration changes the profile of one protein to another [43]. This concept could clearly be applied to CAR class switching, e.g., to change CAR-CD19 to CAR-CD22. When studying this system, it was shown that transient and reversible deactivation of CAR-T prevents the appearance of depletion features and puts cells into a memory cell-like state. Moreover, elimination of the CAR protein also allows for the reversal of epigenetic changes characteristic of already exhausted lymphocytes and enhances their therapeutic effect [42]. Down-regulation of depletion has also been reported after the use of dasatinib [23]. Dasatinib binds to lymphocyte-specific protein tyrosine kinase (LPK), blocking the phosphorylation of CD3z and thereby ablating the CAR signal. This blockade is so effective that dasatinib can be treated as a disabling switch affecting the receptor, but it does not lead to its degradation or transcriptional regulation [44].

A slightly different approach is presented by the SWIFF-CAR mechanism. The CAR is linked to the degron by HCV NS3 protease and to a protein fragment destroyed by HCV NS3 (Figure 2C). This means that after the formation of a common protein chain, the protease cuts the chain into two parts: a fragment containing the CAR and a fragment containing the degron. However, if the protease is stopped by its inhibitor asunepravir (ASN), it leads to the survival of the degron-tagged chain and degradation in its entirety [45].

The small-molecule compound lenalidomide (IMiD) has also been used to control degradation in both off and on switches [46,47]. In both cases, IKZF3 (a degron containing zinc fingers) is attached to the C end of the CAR. In the off switch, administration of IMiD allows recruitment of the E3 complex to the CAR protein. This occurs through binding of IKZF3 to CRBN. CRBN is part of the CRL4^CRBN^ complex, whose attachment leads to CAR protein degradation and deactivation of CAR-T cytotoxic activity (Figure 2D). The on/off switch consists of two components: scFv fused to CD28 and IKZF3 and the CD28 domain fused to CRBN and CD3z. The mechanism of action is based on the dimerization of the IKZF3 molecule with CRBN under the influence of lenalidomide, which allows the signal to be transmitted via CD3z (Figure 2E). In order for this system not to degrade, as in the case of the off switch, IKZF3 was subjected to modifications resulting in the loss of its ability to activate the endogenous E3 complex (amino acids of lysine were replaced by amino acids of arginine), whereas CRBN, which is naturally part of the CRL4^CRBN^ complex, was dissociated from the DDB1 domain [47]. 

### 2.2. Transcriptional Switches

**Figure 3:**
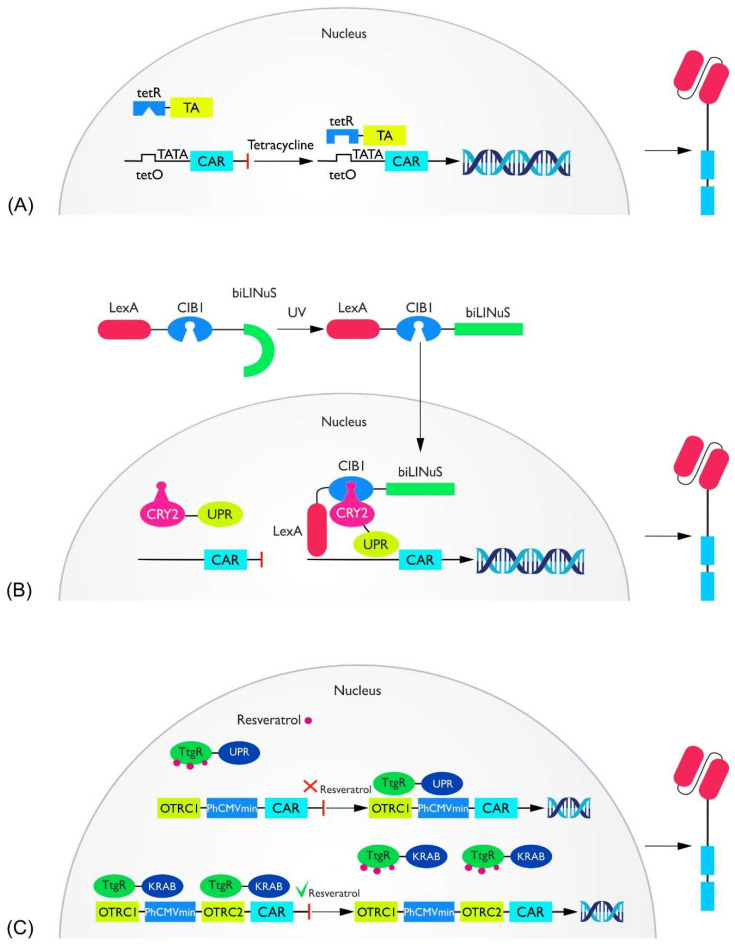
(**A**) The Tet system is activated upon administration of tetracycline. TetR binds to tetO, bringing the TA physically close to the TATA cassette. This activates transcription of the downstream gene—in this case, CAR. (**B**) The molecule composed of CRY2 and UPR is located in the nucleus, while the LexA-CIB1- biLINuS (LCB) molecule is located outside the nucleus. Upon UV delivery, biLINuS, which previously blocks the flow of LCB into the nucleus, changes spatial conformation and allows passage through the membrane. In the nucleus, LCB binds to CRY2-UPR (interaction of CIB1 with CRY2). LexA recognizes the binding site to DNA, and UPR activates transcription of the CAR gene. (**C**) Off switch at the top. When resveratrol is in the environment, it binds to TtgR, which blocks UPR attachment to DNA and activation of transcription. When resveratrol is removed, TtgR binds to Otrc1 and VPR activates PhCMVmin, leading to CAR expression. Off switch at the bottom. When resveratrol is absent, KRAB blocks PhCMV and transcription does not occur. When resveratrol is added, the attachment of TtgR to DNA is blocked, causing KRAB to dissociate (and, consequently, transcription occurs).

(a) Tet system. The tetracycline-induced system is based on prokaryote-derived mechanisms of tetracycline resistance. The system consists of three components. The first is a transcriptional modulator: a tet-sensitive DNA binding domain (tetR) connected to a transcriptional activator (TA). The second is a tetracycline-responsive promoter: tet-R binding sequences (tetO) located above the TATA box. The last is tetracycline (Tc) or a derivative thereof. In the Tet-on system, the addition of tetracycline is followed by its binding to tetR, which causes the entire complex to attach to tetO, which in turn allows TA activation of the promoter (Figure 3A) [48,49]. This system has been used in CAR-T as a doxycycline (Dox)-dependent regulator of CAR transcription targeting CD19, CD147, or CD38. Studies have consistently shown a strong correlation between the presence of Dox and CAR expression, increased proliferation, cytokine production, and cytotoxic effects [50,51,52,53]. The time between Dox administration and the peak of CARs activity ranged from 24 to 48 h (depending on the Dox concentration), similar to the deactivation of CARs after the cessation of Dox administration [52]. Unfortunately, basal Tet system activity is detected independently of Dox administration when using a one-vector system. When the two-vector method is used, this problem does not occur [51]. A solution to the basal activity of the Tet-on system has also been proposed in the CAR context, by introducing the G72V mutation in the TetR region. This resulted in a 40-fold reduction in Dox-independent activation [54]. An interesting study that proposed the possibility of additional control coupled the activation of the Tet system, not only to Tc but also to blue light similar to the Cre system [55].

(b) Light-dependent transcriptional control. The LINTAD system offers molecular control of CAR expression dependent on blue light stimulation. In the absence of blue light, cells remain inactive, whereas after the stimulus, CAR production is activated and persists for approximately two days. The molecular mechanism of this system is based on two physically separated parts: CRY2 linked to VPR (activator of transcription), located in the nucleus, and CIB1 linked to LexA (DNA binding domain) and biLINuS (together, LCB), present in the cytoplasm. When exposed to light, two events take place. First, biLINuS changes its shape, which enables LCB to move into the nucleus. Second, CIB1 dimerizes with CRY2, leading to the binding of LexA to the corresponding DNA fragment and allowing VPR to activate transcription (Figure 3B). In mouse studies, the LINTAD system has been shown to activate cells within the subcutaneous tissue. This system can be modified so that CAR expression is more durable, but this decreases the possibility of controlling therapy [56].

A more complex strategy is presented by the TamPA-Cre system. As in LINTAD, two spatially separated compounds are present; however, in this case, passage to the nucleus of the DNA-binding fragment occurs after the addition of tamoxifen. This is followed by dimerization of nMag-pMag molecules, which leads to transcriptional activation. This system generates an additional CAR-T activation condition that fits into the logic gate strategy to avoid on-target off-tumor attacks [57].

A limitation of systems based on blue and UV light activation is the low penetration depth. In cases of superficial neoplasms such as melanoma, these systems could potentially be applied, although for conditional light-induced activation rather than as an off-switch. Switches, although guaranteed to inactivate surface lymphocytes within melanomas, would not help to silence lymphocytes that are present, for example, in the brain, causing neurotoxicity [58]. An intuitive solution to this problem may be to construct biochemical systems that respond to near-infrared light. Infrared light penetrates deep into tissues, although studies of the skull show that penetration through the skull into the brain appears to be quite shallow, reaching only 3–4 cm, and is so limited that it does not produce biological effects [59]. This could limit the use of LINTAD and TampPA-Cre therapy in the treatment of brain tumors (no possibility of activation). It is worth noting that if the development of biochemical molecules dependent on light of a higher wavelength of molecules would be difficult, it is possible to use UCNPs (upconverting nanoparticles). They have the ability to locally convert infrared light to blue light, a property that eliminates the limitation due to the shallow penetration of UV alone [60]. Moreover, UCNPs can act specifically in the tumor microenvironment. For example, it is possible to enhance bioluminescence if the UCNP environment has a reduced pH [61]. Thus, this system allows not only the control of CAR-T activity, but also enables specific local activation which could help to avoid on-target off-tumor attacks.

(c) Zinc finger protein. A molecule derived from tamoxifen, i.e., 4-hydroxytamoxifen (4-OHT), was also used to create a switch-based transcriptional molecule consisting of the zinc finger protein N1, a modified ligand-binding domain of the estrogen receptor, and the transcriptional activation domain VP64. Activation of CAR transcription occurs upon the addition of 4-OHT and binding of transcription factor to response elements [62].

(d) The RESrep system. The RESrep system is based on a modification of the natural efflux pomp system, TtgABC derived from Pseudomonas putida. RES consists of two components. The first is the ResA3 molecule, composed of TtgR (a resveratrol-dependent activator) linked to a synthetic VPR molecule (concatenated with transcription factors VP64, p65, and Rta) via the C end of TtgR. The second is PResA1, with a resveratrol-dependent promoter—OTRC1, located opposite PhCMVmin (minimal human cytomegalovirus immediate—early promoter) (Figure 3C). Expression of ResA3 is constant, which allows its binding to the promoter and its activation with subsequent protein secretion. However, if resveratrol (a switch control molecule) is present in the environment, it will release ResA3 from the promoter and thus inactivate it. It has been shown that the activated gene was the CD19-CAR construct. The effect appeared to be a reduction in killing, cytokine production, and expression of CD69 (a marker of catharticity). A reverse system was also developed, i.e., induction after resveratrol administration. RESind consists of ResR1 (TtgR fused to the Kruppel-associated box protein C end, TtgR-KRAB) and PResR12 (OTRC1/2 + PhCMV). OTRC1 binds ResR1/2 and surrounds the 3’ and 5’ side of PhCMV. If resveratrol is absent, ResR1 blocks PhCMV by binding to OTRC1 and OTRC2. When resveratrol is administered, ResR1 is released and PhCMV is activated, which is associated with the activation of transcription of the desired gene [63].

## 3. Killing Switches

Killing switches are among the simplest and one of the most effective ways to control CAR-T. Their common feature is to bring about deactivation of CARs by killing. The obvious consequence of this is the inability to continue using the therapy and the need to generate new CARs or to discontinue treatment. Despite this, iCas9-based switches represent a group of already clinically-tested switches in CAR therapy [64].

Undoubtedly, a major advantage of using such systems is the ability to eliminate accidentally transduced, improperly purified, tumor cells that, after acquiring the ability to generate an antigen receptor, become resistant to CAR-T by masking their epitopes [65]. In this case, a non-transformed blast will be killed by CAR action, and an artificially transformed blast will be killed by kill-switch action [66]. This is an example of a unifying application of kill switches not achievable by other methods.

Here, we distinguish three basic groups based on different mechanisms of action: iCas9, ganciclovir-dependent, and ADCC-dependent.

### 3.1. Inducible Caspase-9

**Figure 4:**
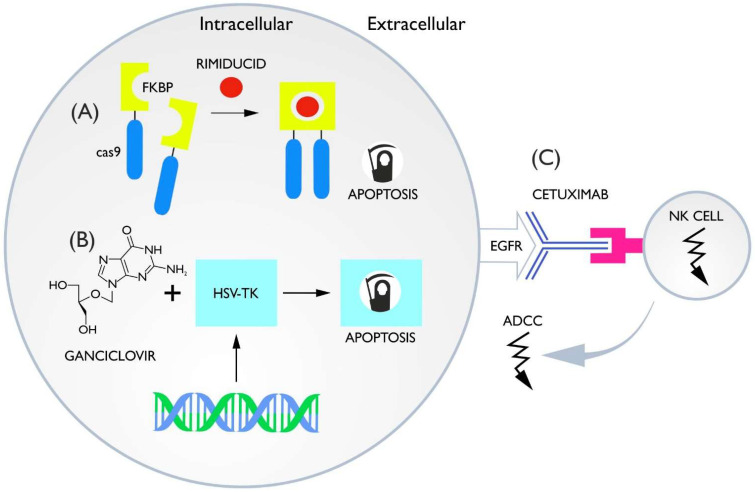
(**A**) Caspase-9 is connected to the homodimerizing protein FKBP. When rimiducid is delivered, homodimerization occurs and caspases send an apoptotic signal to the cell. (**B**) The thymidine kinase (HSV-TK) converts ganciclovir (GCV) into a toxic product and allows selective elimination of TK+ cells in vivo. (**C**) There is a protein on the cell membrane that is a target for the monoclonal antibody. Upon binding of the monoclonal antibody to the epitope, an antibody-dependent cytotoxic effect is induced by NK cells.

The mechanism of action of the iCas9 switch is based on small molecule-dependent homodimerization (AP1903/rimiducid or AP20187). Modified human caspase-9 (lacking the natural dimerization domain) is connected to the FKBP molecule, which enables dimerization and thus activation of caspase-9 under the condition of rimiducid presence (chemical-induced dimerization (CID) occurs) (Figure 4A) [67,68]. These switches are characterized by high efficiency exceeding 85–90% after a single administration of AP1903, which ranks them among the most effective inactivating switches [66,69,70]. A variation of the iCas9 system has also been developed in which the iRC9 system utilizes heterodimerization of the FRB molecule (FKBP-Rap binding) and FKBP12. Such modification enables the creation of an orthogonal switch in which rapamycin is the molecule that activates caspase-9 dimerization (responsible for killing) and rimiducid activates the MyD88 costimulator (described in Section 5) [71]. It is worth mentioning that rapamycin possesses immunosuppressive properties, which may further enhance the potentiation of the switch [72]. The iCas9 system has been successfully applied in clinical trials, not only for T cells but also NK cells and in the laboratory cord-NK cells [73,74].

### 3.2. HSV-TK Ganciclovir

Ganciclovir-activated switches require the introduction of the herpes simplex virus-1 thymidine kinase (HSV-TK) protein into the CAR cells. This enzyme is not expressed in native human cells. Under the influence of HSV-TK, ganciclovir (although acyclovir and penciclovir also work) changes into a toxic metabolite, leading to cell death via a mechanism that is not yet fully understood (Figure 4B) [75]. This system has been tested many times in clinical trials where its safety and efficacy were proven [76]. Currently, an artificial version of HSV-sr39TK is used, having five changed amino acids and characterized by 14-fold smaller Michaelis constants, guaranteeing higher efficiency and safety [77]. In order to achieve the highest killing efficiency with suicide systems, it is necessary to ensure that all lymphocytes possess a killing system, therefore, they must be adequately purified prior to administration. HSV-TK has been designed with cell membrane markers (truncated CD34 or NGFR) allowing magnetic selection and thus enrichment of CAR-T with the desired switch [78,79]. Such methods for verifying switch expression could also be applied to other switches, although switches showing expression on the membrane such as those based on the ADCC effect would not require loading of the vector with additional markers such as intracellular switches.

The use of HSV-TK compared to iCas9 yields similar results, but the disadvantage of this system is the need to store ganciclovir at a highly alkaline pH (11) and HSV-TK immunogenicity [80]. However, an advantage of the ganciclovir-based system is that it allows in vivo PET imaging studies. The HSV-TK gene can be used as a reporter gene to phosphorylate and retain the 18F-labeled compound in the cell (mutant HSV-srTK is more sensitive to 18F). This allows us to monitor the location of CARs in the body and to assess the function of the switch upon activation (a decrease in luminescence will indicate a decrease in cell number) [81,82] since the intensiveness of the PET signal is proportional to HSV-SrTK activity.

### 3.3. ADCC Switches

Switches based on the antibody-dependent cytotoxic effect (ADCC) assume the production of a membrane target that can be detected by an antibody capable of cytotoxic effects, resulting in the death of the labeled cell. Complement-dependent cytotoxicity (CDC) is also involved in cell destruction. Currently, monoclonal antibodies such as anti-EGFR (cetuximab) or anti-CD20 (rituximab) are used (Figure 4C) [83,84]. The ability to kill CAR-T is about 82% and is slightly lower when using cetuximab in relation to iCas9 and HSV-TK switches. Perhaps magnetic immunoselection enrichment procedures would enable higher efficiencies, especially since these switches do not need an additional marker as they are already the target for antibodies. Similar to HSV-TK, ADCC-dependent switches can provide a tracer for in vivo PET, except in this case, antibodies that bind to the antigen on the CAR surface are labeled [85]. Despite these advantages, these systems are associated with some limitations. First, it is worth noting that the penetration of cetuximab as well as rituximab through the blood–brain barrier (BBB) is very low [86,87]. This may be clinically relevant given the potentially important role of CARs penetrating the central nervous system in CRS and immune cell-associated neurotoxicity syndrome (ICANS) [88,89]. Moreover, EGFR represents an attractive target for many cancers, and CAR-T therapies of this type are currently being tested [90,91]. The use of an EGFR-based switch eliminates the possibility of using cetuximab as a combination therapy or anti-EGFR CAR-Ts because it would lead to the destruction of CAR-Ts early in therapy. A similar problem also exists in CD-20 [92].

## 4. Adapter Switches

**Figure 5:**
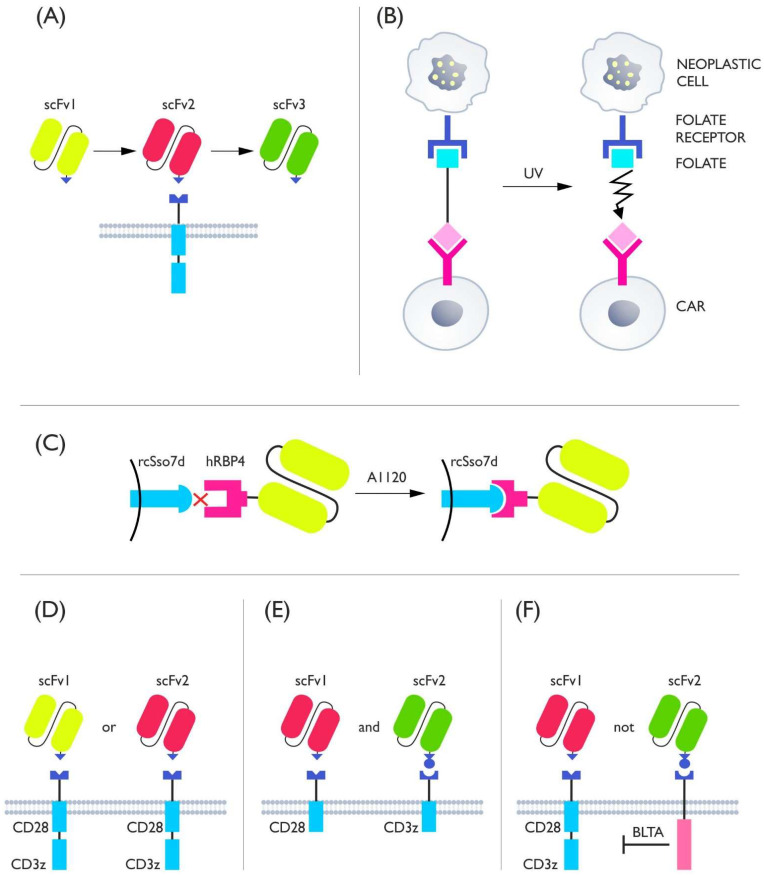
(**A**) Schematic of the universal receptor idea. One CAR is able to bind multiple mediators having identical endings but different binding domains. In this system, CAR does not directly recognize the tumor antigen—it does so indirectly via the appropriate trans molecule. (**B**) The mediator consists of FITC which is the target for CAR and folate which binds to its receptor on the tumor. The mediator is connected by a chemical bond that leads to the degradation of the TM and inactivation of CAR upon UV delivery. (**C**) CAR has a rcSso7d domain that does not naturally bind to hRBP4, which is a component of the TM. When A1120 is added, the conformation of hRBP4 is changed so that it binds to hRBP4 on CAR allowing for a cytotoxic effect. (**D**) OR gate: one type of CAR supplied with two (or more) mediators; one receptor binds many different types of TMs. (**E**) AND gate: CAR splits into a domain containing CD28 (costimulatory part) and CD3z (signaling domain); both receptors need to be stimulated for the cell to be activated, and both receptors have different binding abilities for TM which means that different mediators are needed to activate them. (**F**) NOT gate: if a second signal is recognized, BLTA blocks activation of the stimulating receptor, and the two receptors have different binding abilities to the TM which makes them require different mediators and antigens for activation.

The idea of adapter switches is based on the use of a target molecule (TM), which acts as an adapter binding to CAR on one side and to the target on the tumor cell on the other side. For adapter CAR activation, two events are required: binding of the TM to the CAR on lymphocyte and the binding of the TM to the antigen on tumor cell. Then, an immune synapse is formed, allowing for the cytotoxic effect. This system is called UniCAR because one type of CAR cell can universally bind to many different targets if the appropriate TM is provided [93,94] (Figure 5A). The adaptors can be scFv-based particles or small-molecule compounds that bind to the folic acid receptor [94,95].

The mechanisms that are used in adapter binding to CAR include the leucine zipper (SUPRA CAR), monomeric streptavidin 2 (mSA2) biotin-binding domain, anti-fluorescein isothiocyanate (FITC), biotin-binding immunoreceptor, and various types of scFv, e.g., anti-peptide neoepitope (PNE) or anti-E5B9 [96,97,98,99,100,101]. On the opposite side, TM binds to the tumor cell itself via scFv or, more rarely, via the folic acid receptor.

The use of adaptor switches is associated with several advantages. First, control of CAR activity is accomplished by either entering or withholding TM delivery. This means that the system has the ability to switch CAR both on and off. TMs have a short half-life (due primarily to renal filtration) depending on the type of adaptor and route of administration—intraperitoneal (i.p.) injection extends the half-life compared to intravenous (i.v.). Usually, the half-life ranges from about 30 to 90 min; however, the cp-Fab system reached a 16-hour half-life. Thus, the time required to deactivate CAR is derived from the time the body takes to remove the TM [95,102,103,104]. This means that fast degradation allows a potentially quicker deactivation of CAR, but also requires a more intensive supply of TM. In order to not wait for the spontaneous elimination of the adapter, B. Zhang et al. presented a system composed of a UV light-sensitive (365 nm), small molecule adaptor. This TM consists of three parts: FITC that binds to the ani-FITC CAR on the T cell; a switch part, ortho-nitro-benzyl ester, that is susceptible to UV cleavage; and folate that binds to the folate receptor (FR) on the tumor cell (Figure 5B). To deactivate CAR-T, UV light must be delivered to break the mediator into two parts and prevent T cells from acting. In a study on mice, inhibition of cytokine-induced toxicity was observed within a very short time after UV light treatment. If resumption of CAR-T action is desired, all that is needed is to add the mediator again [105].

Since the need for continuous adaptor infusion due to the short half-life is a clinical burden, attempts have been made to extend the half-life of TM. One of these attempts resulted in the development of the αSTn-IgG4 adaptor. Compared to classical TMs based on scFv, the hinge region and Fc of the IgG4 antibody were additionally included, thus increasing its molecular weight. This achieved a significant (more than 10-fold) extended half-life resulting from the longer settling time of the adaptor in the tumor and slower renal clearance [106].

A similar concept was attempted by adding three 4-1BB domains connected with flexible glycine-serine linkers to TM. As expected, the adaptor half-life was extended. However, in this case, additional costimulation in trans, resulting from the function of 4-1BB, was obtained, which translated into higher CAR expansion. Unfortunately, at the same time, the appearance of spherical obstruction impaired immune synapse formation, which decreased the efficacy of the cytotoxic effect by 10-fold [107].

Ch. Zajc et al. presented an even more complex switch responsible for controlling TM binding through an additional small-molecule compound A1120 (oral drug). The A1120 molecule enables a conformational change in human retinol binding protein 4 (hRBP4) that makes it 550-fold more able to bind to the artificially created scaffold rcSso7d, and chemical-induced dimerization (CID) occurs. The rcSso7d molecule (RS3 mutation) is attached to CAR via the IgG1-Fc spacer domain, while hRBP4, which forms the mediator, is attached (also via IgG1-Fc) to scFv. This design makes it necessary to provide both TM and A1120 to activate CAR; if one of these compounds is not present, as studies have shown, CAR-T will not be active either (Figure 5C) [108]. It is also noteworthy that CAR activation is proportionally dependent on the TM dose (dose-dependent manner), allowing the strength of CARs to be controlled by mediator titration [102].

Another aspect offered by adapter switches is the possibility of CAR-T retargeting. With classical CARs, if antigen escape occurs then therapy usually fails [109]. The UniCAR system allows switching the target to another antigen by changing the adapter [110].

The TM systems have also been successfully adapted to create a logic gate. Logic gates are a strategy of CAR to minimize on-target off-tumor effects by causing CAR to be turned off or on depending on the external signals detected by the T lymphocyte, thus increasing its specificity [111]. In the case of the SUPRA CAR system, TM-based effects have been demonstrated on a large group of different cells: macrophages, NK cells, cytotoxic lymphocytes, and regulatory lymphocytes as well as Th1, Th2, and γδ cells [112]. The simplest OR gate design using the universal CAR system requires only the addition of two different mediators. This leads to CAR activation if only one of the mediators binds to the tumor cell (Figure 5D). The AND gate is slightly more complicated. It requires the separation of the CAR complex consisting of CD3ζ and CD28/4-1BB into two separate receptors that have different binding abilities to TMs, e.g., FOS zipCAR and RR zipCAR. TMs having different binding abilities to CAR and specificity to different antigens on the tumor must be administered simultaneously. This ensures that the lymphocyte will only be activated when the TMs bind to two different antigens (Figure 5E) [96]. A NOT gate has also been developed on the model of the AND gate. As in AND, there is a FOS zipCAR and RR zipCAR but one of the receptors contains both CD3ζ and CD28/4-1BB and the other contains a BLTA domain that inhibits CAR (Figure 5F). The most complex is a three-input gate incorporating an AND and a NOT gate, providing a potentially greater degree of control. Furthermore, gates based on the interaction of many different cells armed with the SUPRA CAR system have been developed, offering the possibility of, among others, changing the polarity of macrophages or silencing TCD4+ by regulatory lymphocytes [112]. As it can be seen, adapter receptor-based systems offer control of CAR activity on many levels, including logic gate operation.

## 5. Costimulatory Switches

Costimulatory switches represent the smallest group of those described in this article. They do not allow the complete abolishment of the action of CAR-T but only the reduction in additional stimulation by manipulating the control molecule concentration. Nevertheless, their applications can be broad since many CAR strategies include enhancing expansion and anti-cancer effects by introducing active costimulatory molecules such as GITR or the KIRS2 protein or enabling endogenous production of interleukins [113,114,115]. Switches that provide regulated costimulation could potentially provide more precise control of CAR and simultaneously help overcome tumor immunosuppression.

**Figure 6:**
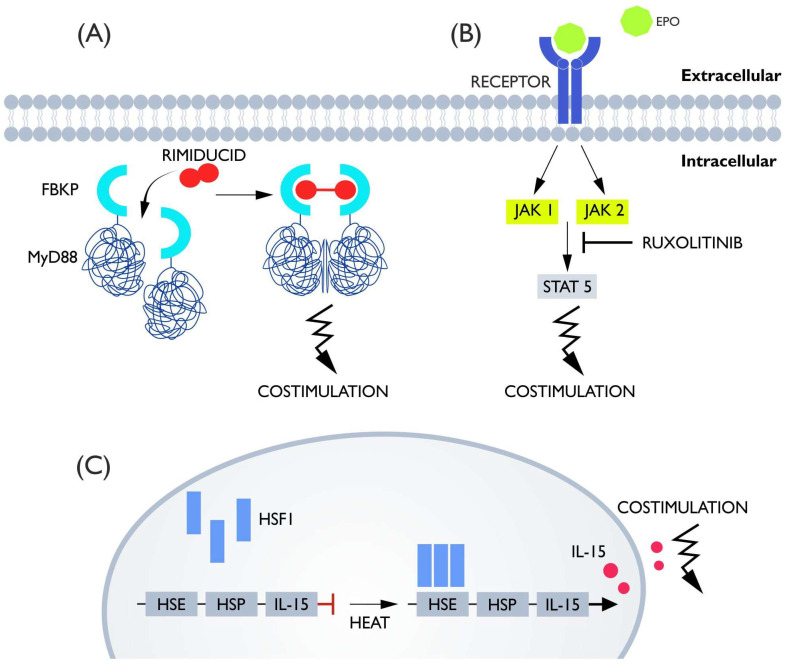
(**A**) MyD88 is activated by a dimerization mechanism. It is connected to FKBP. After administration of rimiducid, FKBP homodimerizes and with it, MyD88, providing an activating signal. (**B**) Erythropoietin receptor-based switch. The receptor for EPO is located on the membrane and provides an activating signal when erythropoietin is detected. This switch can be turned off by administering ruxolitinib which inhibits STAT5 phosphorylation or by restricting EPO. (**C**) HSF1 is, by default, in the free state. When heat is applied it trimerizes and binds to HSE which activates IL-15 gene transcription.

Some of the costimulatory switches are similar to the iCas9 system and based on chemical-induced dimerization. Since the dimerization mechanism can be achieved by multiple methods, this strategy seems to be potentially amenable to modifications in terms of introducing several CID switches into a cell at once, or by testing the coupling of new costimulators to CID domains [116]. Already used costimulatory domains include MyD88 and CD40. In this case, FKBP12_v36_ proteins that were fused to MyD88 and CD40 were used. Analogous to the iCas9 systems, the administration of rimiducid causes homodimerization of FKBP and activation of MyD88/CD40 as a consequence of bringing these proteins together (Figure 6A) [71,117]. The used costimulatory molecules both occur naturally on lymphocytes. MyD88 is a component of the TLR receptor responsible for activation, and CD40 is a well-known costimulatory molecule with a role in defense against T-cell exhaustion, and both act on cells through the NF-kB pathway [118,119]. Studies have shown that continuous activation of MyD88/CD40 positively affects CAR-T activity, with MyD88 much more potent than CD40. Proliferation, survival, and anti-cancer activity were increased. Moreover, increased IL-2 secretion and decreased PD-1 expression were detected. Unfortunately, the presence of more unfavorable cytokines such as IFNs, TNF-a, and IL-6 causing toxicity was also detected, which justifies the introduction of a control mechanism that can limit the potential side effects of additional costimulation [120,121]. The MyD88 and CD40 signaling molecules, in combination with the CID binding domain, allowed the formation of a functional switch regulated by CID infusion. Experiments conducted on anti-HER-2 CAR-T showed that upon the addition of CID, proliferation, cytokine secretion, and killing capacity were significantly higher compared to CAR-T without iCO and to CAR-T iCO but without CID. In the xenograft mouse model, the difference was smaller but still in favor of CAR-T iCO + CID. Moreover, the increase in activity was dose-dependent, which, in contrast to the continuously active MyD88 and CD40, allows for adjustment of CAR-T potency [71,117]. Interestingly, one study introduced a rapamycin-induced caspase-9-based safety switch (iRC9) in addition to iCO, which is described in the section on induced apoptosis. It has been demonstrated that these switches can be simultaneously present in the cell without affecting its molecular functions [71]. This system has also been successfully tested in CAR-NK cells [122].

The second type of costimulatory switch is the artificially-introduced erythropoietin receptor (native and truncated version—EpoRm). The erythropoietin receptor, after binding its ligand erythropoietin (Epo), activates a signaling pathway based on STAT5 phosphorylation via JAK1/2 kinase. As shown in the experiment, CAR-Ts equipped with EpoRm exhibited a higher killing capacity and longer survival than CARs not armed with the erythropoietin receptor. Interestingly, the concentration of Epo naturally present in the body is already sufficient for a positive effect, but the additional use of erythropoietin further enhances the costimulatory effect. In in vitro studies, the concentration of 10 IU/mL allowed resignation from the administration of IL-2, which proves the effectiveness of this solution and makes it possible to consider whether Epo would be a better stimulator than IL-2, which is known to cause toxicity [123,124]. Moreover, the interaction of Epo and IL-2 showed a synergistic effect, and the control of CAR activity can be achieved by ruxolitinib (Figure 6B). This drug, as a JAK1/2 inhibitor, allows inhibition of the costimulatory signal coming from EpoRm without impairing CAR survival for at least two days [123].

Another way to modify costimulation in CAR is a heat-activation-based switch designed by Miller et al. This system is a modified version of the heat shock protein system. When exposed to heat, the heat-sensitive molecule HSF1 trimerizes and binds to HSE, activating transcription of HSP and the target protein (Figure 6C). Naturally, this system can be activated, not only by temperature, but also by hypoxia or mechanical stress. To eliminate this problem, an artificial version of HSE has been created that responds only to heat. The effector genes, which in the case of CAR-T, provide the desired effect, were placed in the region of the activated promoter. The gene tested was IL-15 superagonist (IL-15 SA). The study showed that heat (40–42 °C) followed the expression of IL-15, which significantly increased tumor suppression efficiency and the survival of mice. An important aspect is that the temperature required for activation is both high enough not to be reached in the case of CRS and low enough not to directly destroy the lymphocytes themselves. The study used gold rods as the heat source, which converted infrared light into thermal energy. One of the problems this therapy may encounter is (as with light-based systems) the problem of delivering heat to greater depths. In addition, it should be noted that for consistent activation of transcription, heating once is not enough—heat must be delivered on average every 3–4 days to achieve consistent expression [125].

## 6. Allogeneic Adoptive Therapies

The switches described in this article are often characterized by high complexity. For this reason, their mass production may be hampered if CAR-Ts are applied for autologous use only. The development of allogeneic “off-the-shelf” CAR-Ts would enable much easier installation of complex switch systems.

The primary problem encountered when attempting allogenic CAR-T transfer is graft-versus-host disease (GVHD). From an immunological point of view, the TCR receptor present on αβ T cells is responsible for GVHD. The answer is to inactivate the TCR receptor on the lymphocytes undergoing modification. This can be achieved by using a site-specific nuclease that inserts a CAR construct in place of the TCR receptor or by using a TALEN-based or CRISPR knock-out [126,127]. The costimulatory domain has also been shown to influence risk: 4-1BB increases the risk of GVHD compared to the CD28 domain [128]. Of course, it is also possible to use cells that do not naturally possess the TCR receptor such as NK cells or γδ T cells, which have a different TCR structure; such cells are less likely to cause GVHD [74,129].

The second obstacle is the elimination of allogeneic CAR cells under the influence of the host immune system. Here, there are also several methods. It is possible to decrease host lymphocytes before they eliminate CAR; for this purpose, CD52, which is the target for alemtuzumab, should be knocked out (CRISPR) in CAR-T. The administration of anti-CD52 Ig will eliminate T cells except those that do not have CD52 and thus CAR-T, and this can be realized simultaneously with the elimination of TCR in a single action to prevent GVHD [126]. Another method is to knock out the MHC itself, which makes the target of the TCR receptor cease to exist and CAR-Ts are not attacked by host T cells. Unfortunately, they also become vulnerable to NK cell attacks [130]. To avoid being killed by NK cells, further modifications must be made. One possibility is to use NK inhibitory ligands, e.g., CD47, PD-L1, or HLA-E, on the CAR membrane. Another option may be to knock out HLA -A/B while leaving HLA-C, this allows for easier compatibility matching in the HLA system (12 alleles of HLA-C allow for matching in 95% of the world population) while blocking NK cells [131,132,133].

## 7. Conclusions

The switches used in CARs are a heterogeneous group of solutions that share the ability to remotely control the activity of chimeric antigen receptor lymphocytes. The following classes of switches are proposed: receptor switches, killing switches, mediator switches, and costimulatory switches. This classification was established based on the mechanism of action to facilitate the design of similar solutions in the future. It seems that no single solution is unequivocally better than another because of the complex properties they possess.

The greatest advantage of receptor and mediator switches is the high control of CAR-T activity while not having to kill them. When deciding to install one of the two aforementioned switches, the most important question seems to be whether we can construct continuous TM infusions. If so, the high plasticity of mediator switches seems to be a significant advantage, especially if attempts to create renal filtration-resistant TM are successful. On the other hand, receptor regulatory switches could also be applied to remotely controlled multi-specific CARs if using degron-based systems. This means that two evolutionary branches may arise on the way to achieving antigen-switching CAR. 

Costimulatory switches may not be used alone, but their positive costimulatory effect seems to have many applications. If methods could be developed to accurately assess the risk of the CRS onset in real-time, perhaps subtle modulation of CAR activity by costimulatory switches would allow lymphocytes to be tuned to the required situation, without the need for radical deactivation.

Against the backdrop of these promising approaches, the strategy of irreversibly killing cells with killing switches seems to pale somewhat, although it should be remembered that clinical trials are currently being conducted on these very mechanisms, which seem to be the most reliable due to their simplicity. Perhaps killing switches are required in switch development as a temporary, interim, ad hoc measure before better solutions are refined. Due to many complex aspects of their operation, the choice of the most advantageous solution may not be obvious.

One of the biggest issues of CAR T-cell design and production is the selection of a suitable system that will introduce the CAR construct into the cells. The two most commonly used options for CAR T cell generation are viral-based vectors or nonviral vectors. Many details (pros and cons) should be considered with both scenarios (viral and nonviral). Moreover, within these systems, important differences should be also recognized. Not all viral systems are accurate. For example, adenoviral systems are not. In the case of CAR-T, the temporary presence of a transgene is not sufficient, and in vivo re-administration is impossible, whereas adenoviral vectors are not able to efficiently integrate genomes [134]. Required integration brings issues; chromosomal integration may be associated with oncogenesis, especially if the DNA vector disrupts proto-oncogenes or tumor suppressor genes [135]. Unfortunately, gamma retroviral vectors usually integrate into gene promoters, resulting in higher oncogenic danger [136]. The improved biological safety of lentiviral vectors is due to safer localizations of transgenes after incorporation [137]. Described dangers and DNA size limits bring attention to the nonviral vectors. The systems presented in this article require the incorporation of more than one transgene, and the enlarged size of incorporated DNA can be a factor favoring nonviral vectors. Moreover, the number of infections and selections can be constrained by a limited number of possible resistance drugs. The PiggyBac transposon system was proven to be able to transfer multiple genes into T cells [138]. Unfortunately, nonviral systems show low transfection efficiency. A classic example is the Sleeping Beauty system. Researchers have gradually solved the problem of the lower integration efficiency of this system [139]. Another transposons system, Tol2, offers the possibility to deliver even greater fragments of DNA (100-200 kb) than Sleeping Beauty [140]. However, the best alternative to the Sleeping Beauty system can be the PiggyBac transposon system [141]. Lately, hybrid viral/nonviral systems have also been proposed [142].

Transposon-derived plasmid vectors depend on chemical on physical methods of delivery (electroporation, lipid nanoparticles), which makes their use more complicated. It is difficult to determine which system, viral or nonviral, will become more useful during CAR-T cells engineering, but the growing number of required modifications makes transposon systems quite attractive.
